# Supplementary material for: Future changes in the trading of virtual water
Source: Nat Commun. 2020 Jul 20;11:3632. doi: 10.1038/s41467-020-17400-4 (PMC7371698; doi:10.1038/s41467-020-17400-4)
Supplement: Supplementary file 1 — Supplementary Information [file 41467_2020_17400_MOESM1_ESM.pdf]

Supplementary Information for:

"Future changes in the trading of virtual water"

Neal T. Graham[1,2,3], Mohamad I. Hejazi[1, 3], Son H. Kim[1], Evan G. R. Davies[4], James A. Edmonds[1], Fernando Miralles-Wilhelm[1, 2, 3, 5]

[1] Joint Global Change Research Institute, Pacific Northwest National Laboratory, College Park, MD
[2] Department of Atmospheric and Oceanic Sciences, University of Maryland, College Park, MD
[3] Earth System Science Interdisciplinary Center, College Park, MD
[4] Department of Civil and Environmental Engineering, University of Alberta
[5] The Nature Conservancy, Arlington, VA

Author correspondence to Neal Graham, neal.graham@pnnl.gov

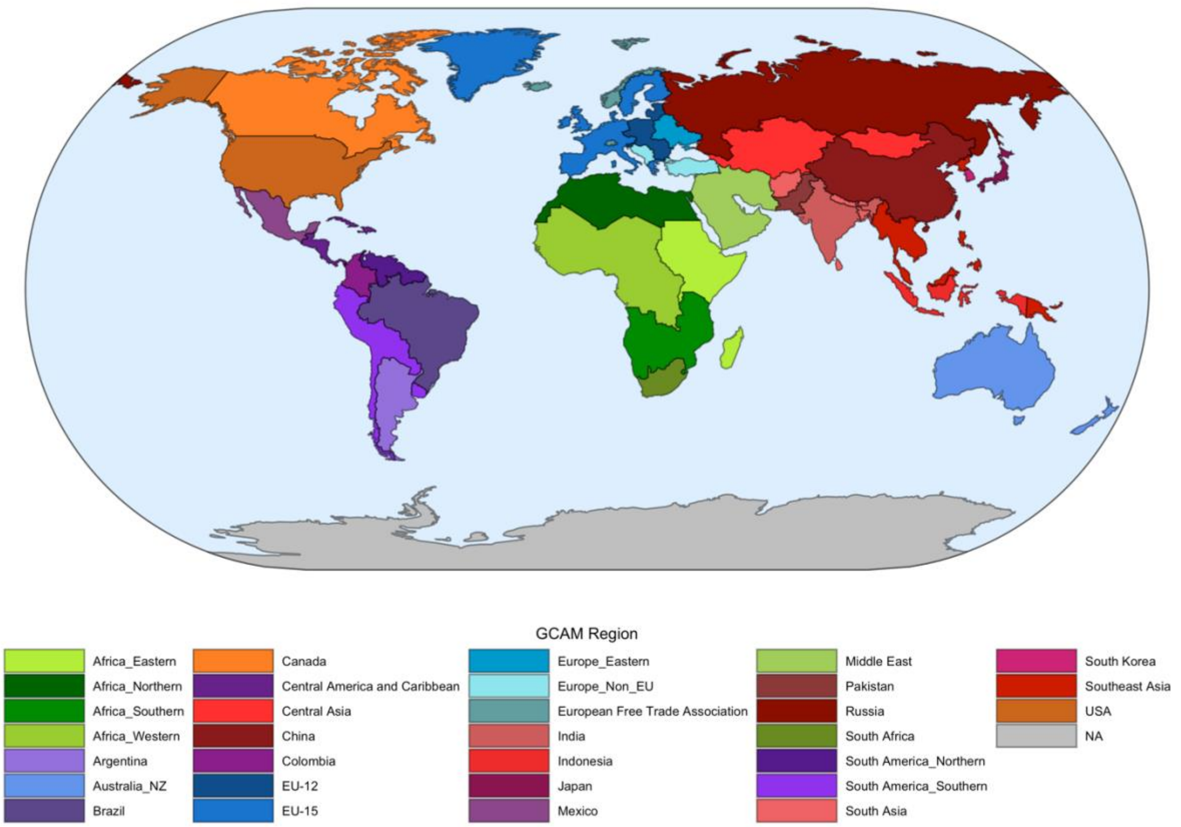

Supplementary Figure 1| GCAM energy-economy regions. Representation of each of the 32 energy-economy regions in GCAM. Demand is modeled at this resolution and trade is downscaled from here using the basin level production to regional production ratio of each GCAM region. Antarctica is not a region where production and demands are tracked and therefore is classified as NA.

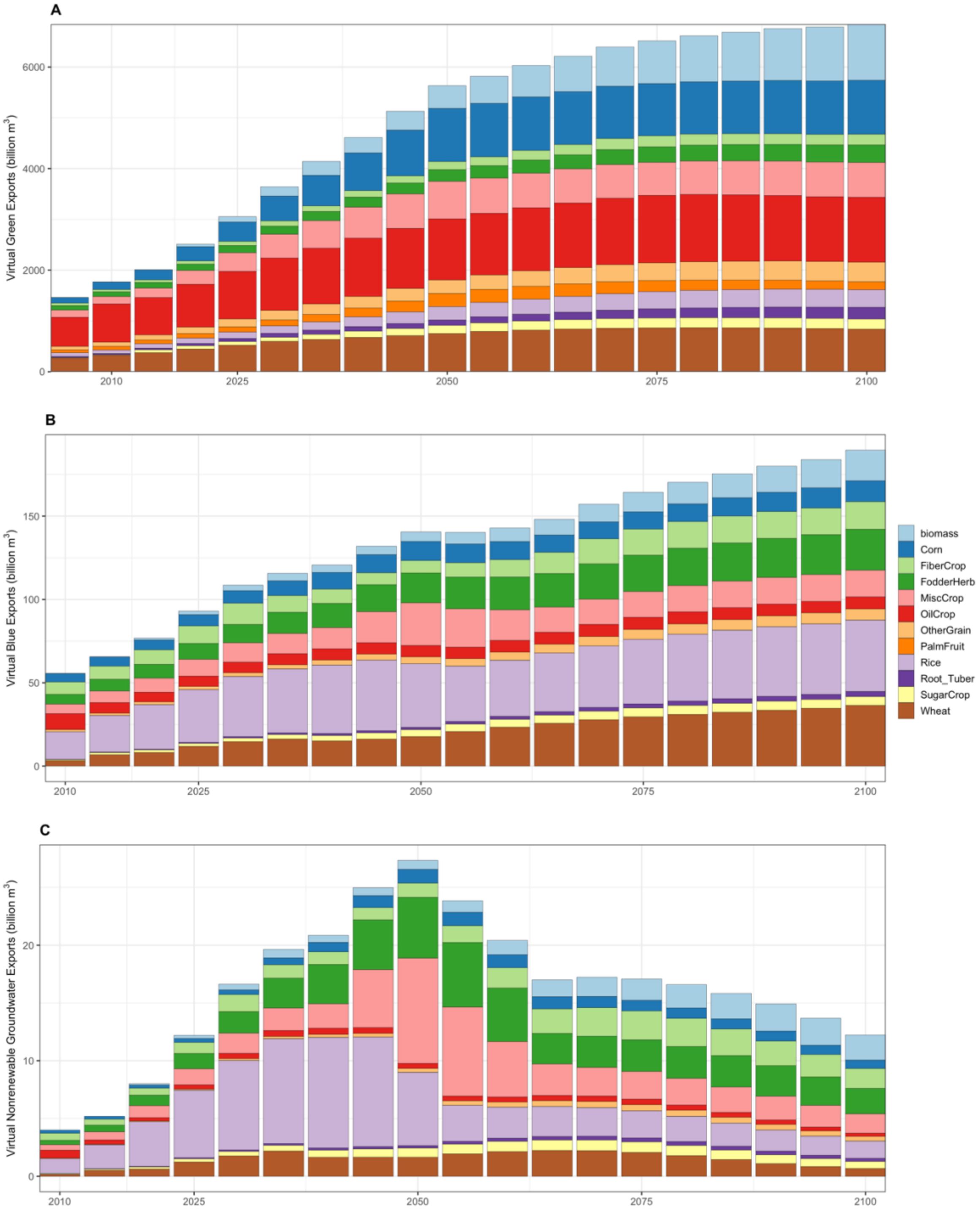

Supplementary Figure 2| Crop breakdown of each virtual water trade component averaged across the SSP2-RCP6.0 GCM scenarios (*n*=5). A, Virtual green water exports by crop, from 2010 to 2100. An intensification of every crop type is seen throughout the century. B, Virtual blue water exports by crop and region. Increases in wheat and rice make up the largest portion of virtual blue water exports. C, Virtual groundwater exports by crop.

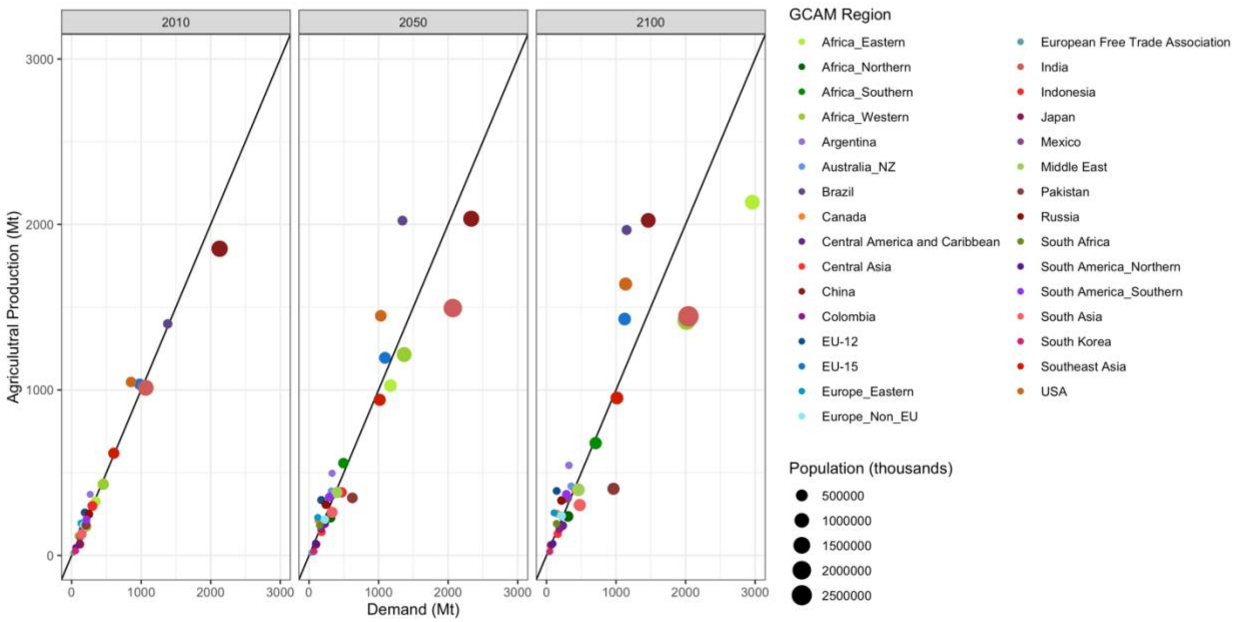

Supplementary Figure 3| Demand and Production comparison. Production deficits and surpluses for each region (Mt). Regions falling below the one-to-one line represent regions in which domestic production is not sufficient to meet demands, which above the line show regions in which excess production can be exported. Population size (thousands of people) is shown as the size of individual points, with larger points located in areas of high demand. China moves from deficit to surplus by 2100 with little overall change in production values. India, Africa Western and Africa Eastern show large increases in demands and production, but each region is in a significant deficit, leading to large amounts of imports needed to meet demands.

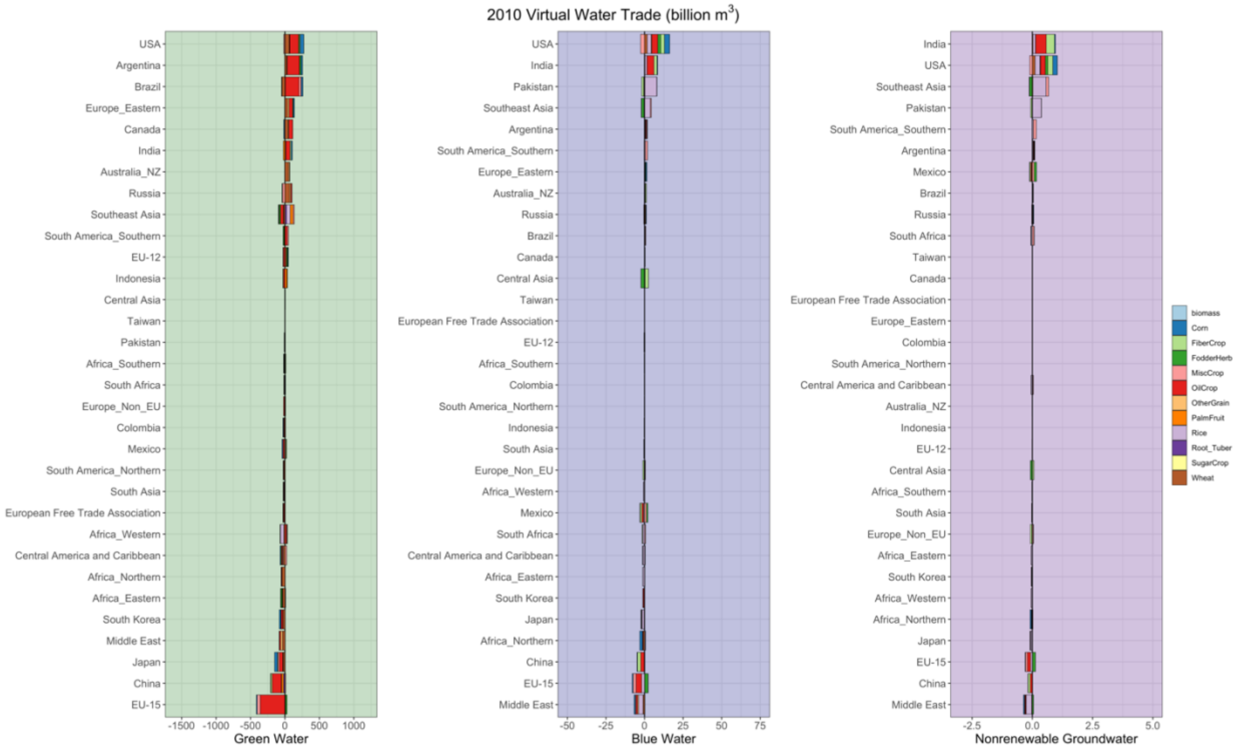

Supplementary Figure 4| Virtual water trade fluxes by crop and aggregate GCAM region in 2010 for all SSP2-RCP6.0 GCM scenarios (*n* = 5).

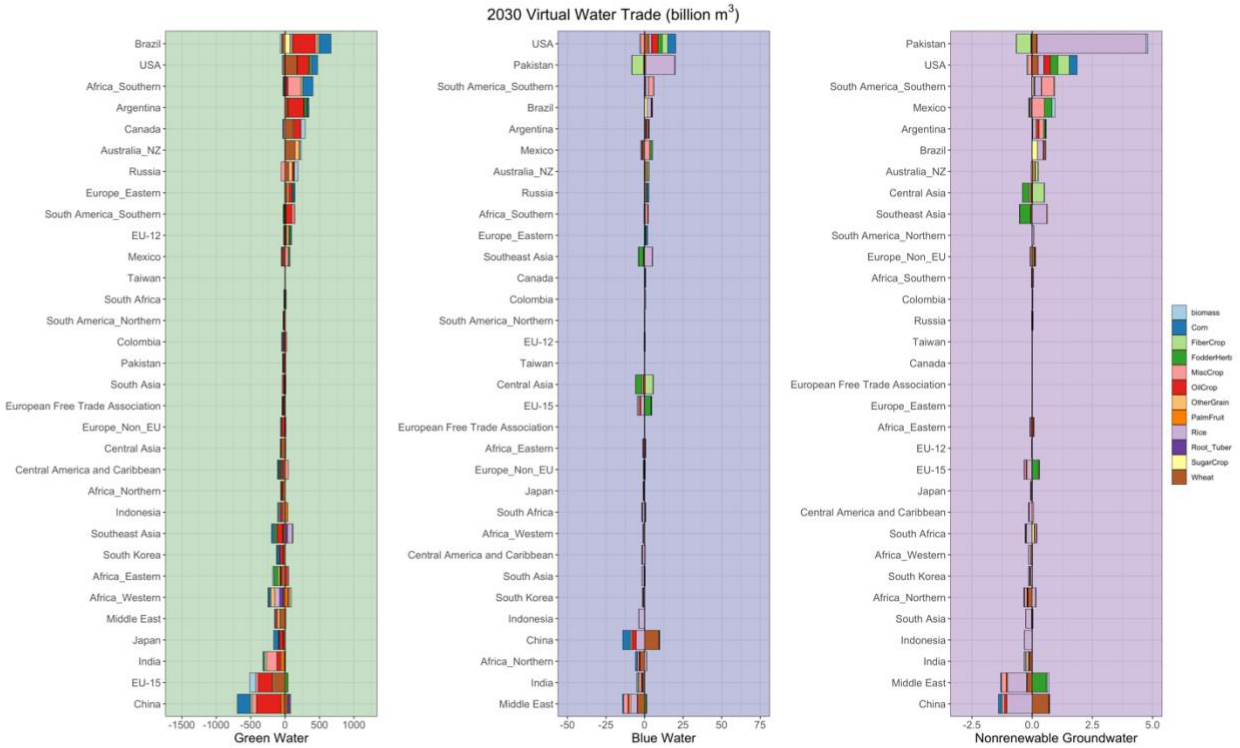

Supplementary Figure 5| Virtual water trade fluxes by crop and aggregate GCAM region in 2030 for all SSP2-RCP6.0 GCM scenarios (*n* = 5).

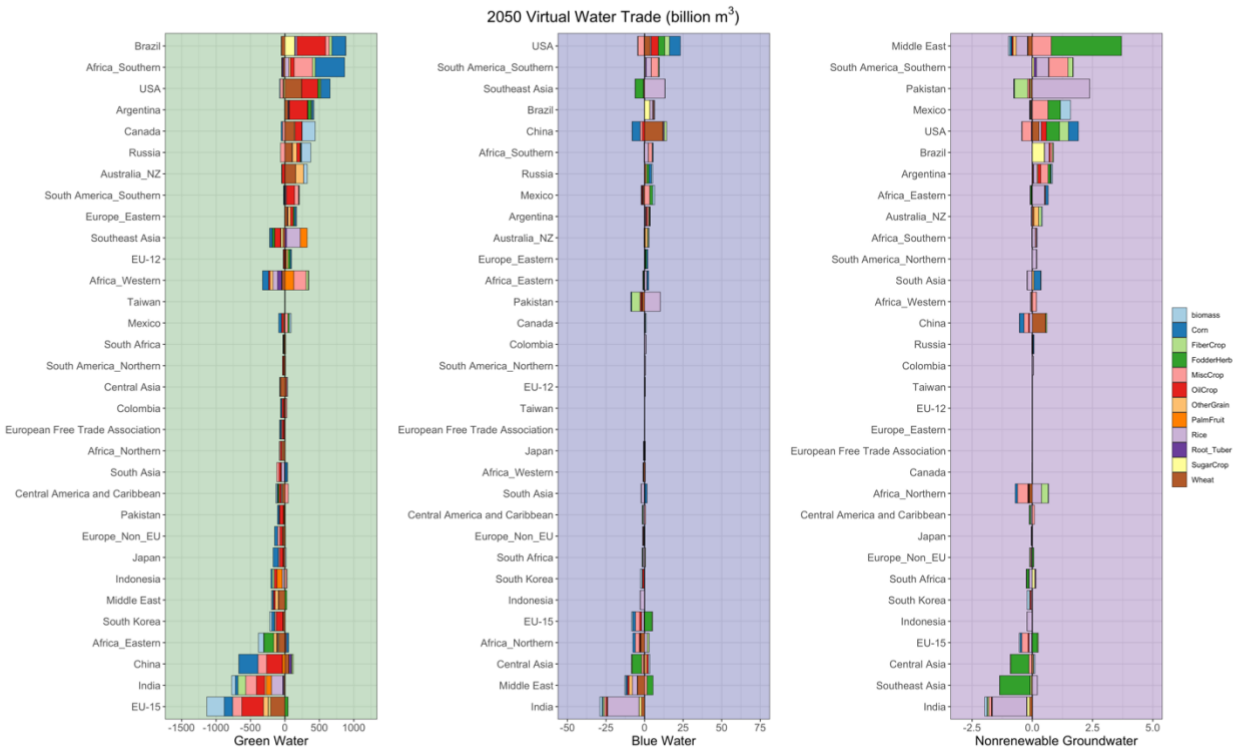

Supplementary Figure 6| Virtual water trade fluxes by crop and aggregate GCAM region in 2050 for all SSP2-RCP6.0 GCM scenarios (*n* = 5).

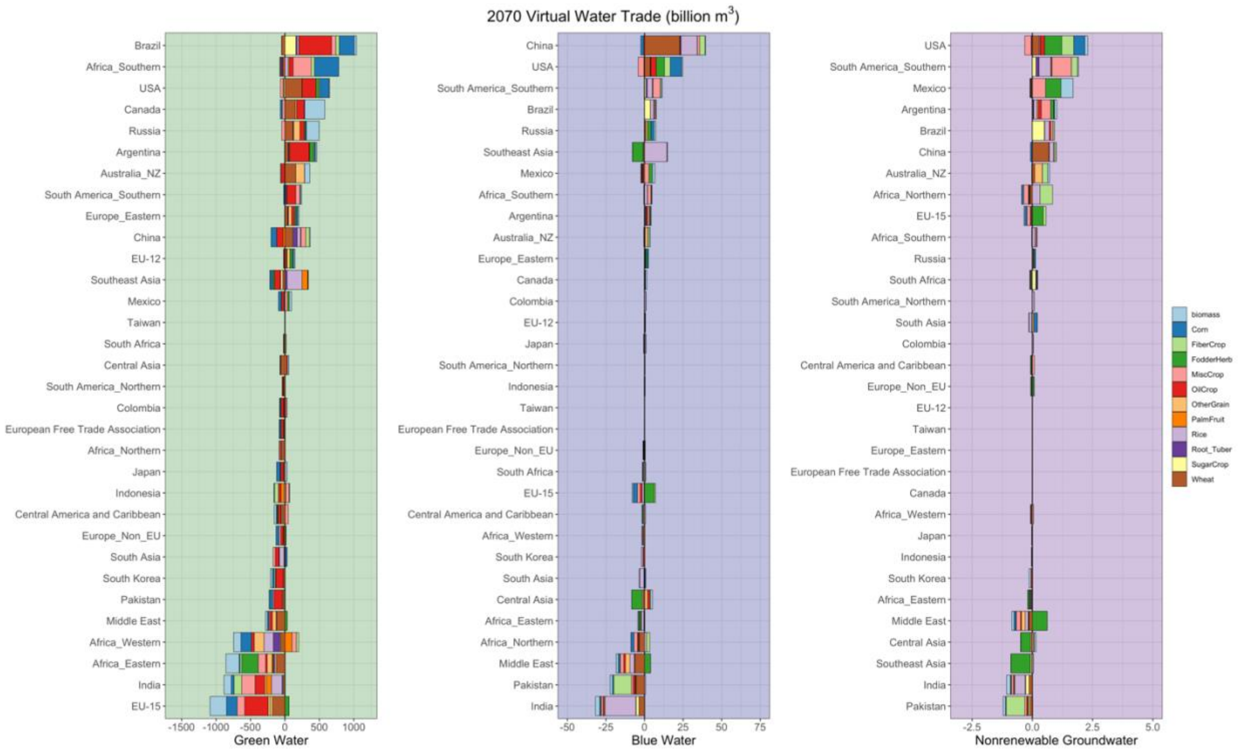

Supplementary Figure 7| Virtual water trade fluxes by crop and aggregate GCAM region in 2070 for all SSP2-RCP6.0 GCM scenarios (*n* = 5).

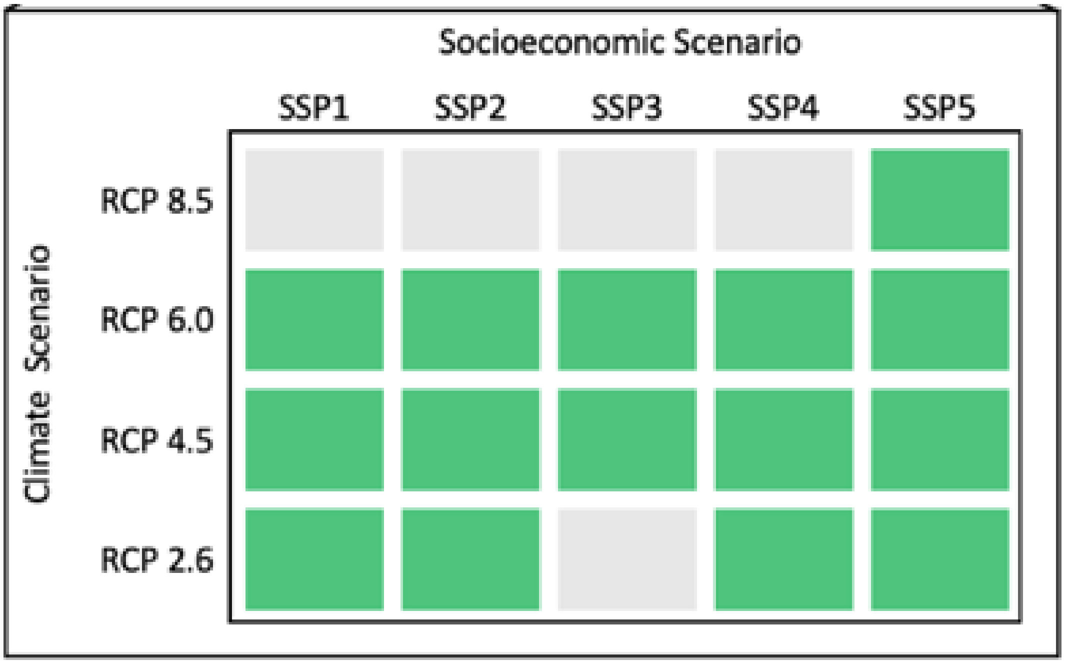

Supplementary Figure 8| from Graham et al., 2020 SSP-RCP scenario matrix of plausible socioeconomic-climate futures available in GCAM (Calvin et al., 2017; Graham et al., 2020).

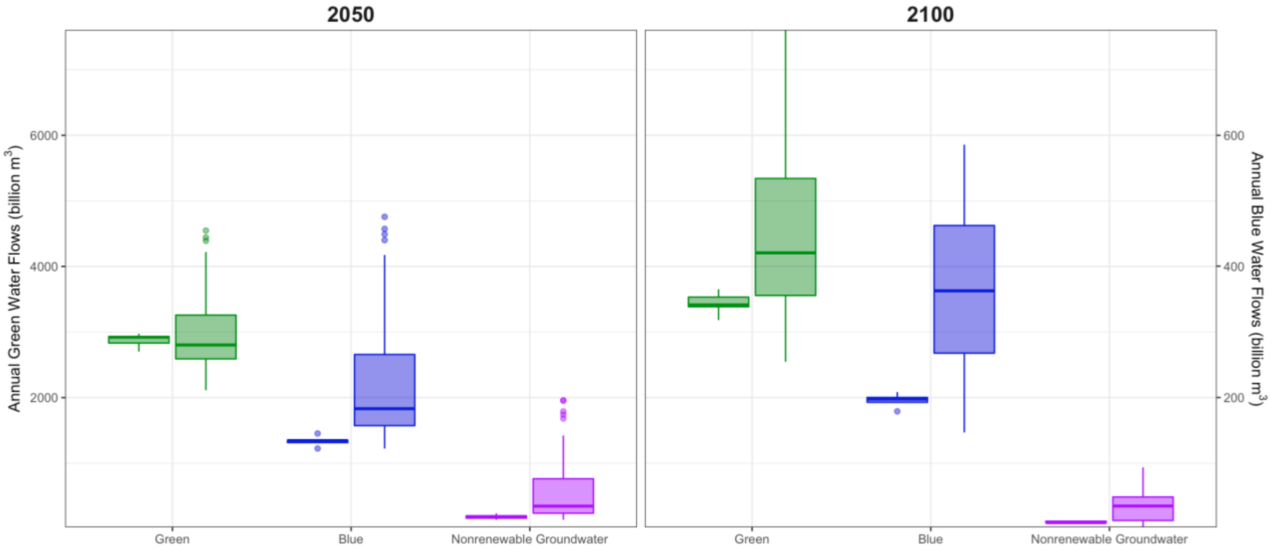

Supplementary Figure 9| Boxplot uncertainty range for green, blue, and nonrenewable groundwater trade in 2050 and 2100. The major y-axis (left) represents green water trade, while the minor (right) represents both blue and nonrenewable trade. In each pairing, the left boxplot represents the range surrounding the SSP2-RCP6.0 scenarios (*n* = 6) investigated in the main text, whereas the right boxplots represent the full suite of SSP-RCP scenarios depicted in Supplementary Fig 8 (*n* = 90). Uncertainty range is shown across all SSP-RCP combinations currently plausible in GCAM (Calvin et al., 2017; Graham et al., 2020). Boxplots shown incorporate maxima and minima quartiles as 25% and 75% respectively, while center is median value of all observations. Outliers, represented as points, are greater than 1.5 * IQR or less than -1.5 * IQR.

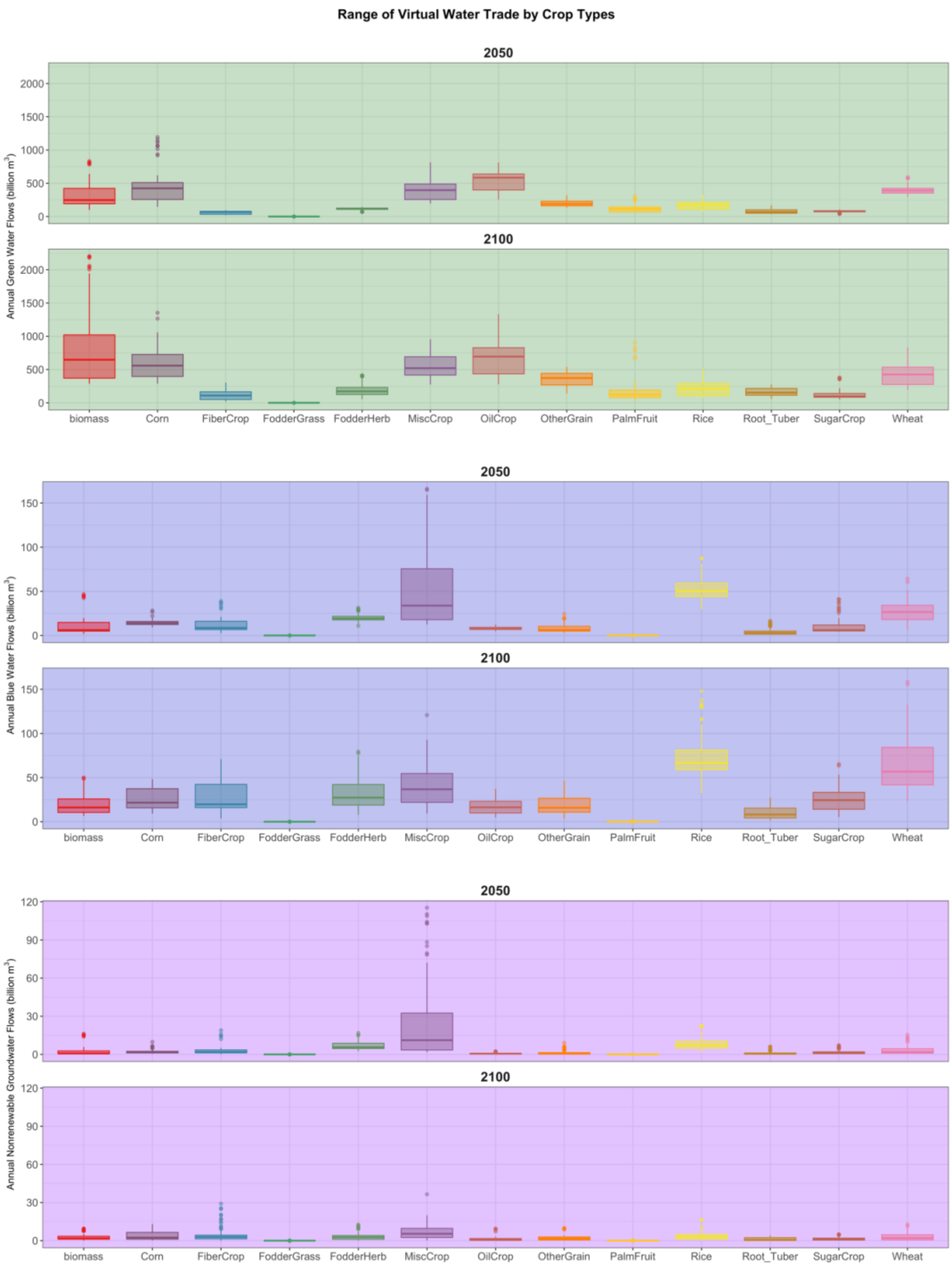

Supplementary Figure 10| Boxplot uncertainty range by crop type for green, blue, and nonrenewable groundwater trade in 2050 and 2100. Uncertainty range is shown across all SSP-RCP combinations currently plausible in GCAM (n = 90) (Calvin et al., 2017; Graham et al., 2020). Boxplots shown incorporate maxima and minima quartiles as 25% and 75% respectively, while center is median value of all observations. Outliers, represented as points, are greater than 1.5 * IQR or less than -1.5 * IQR.

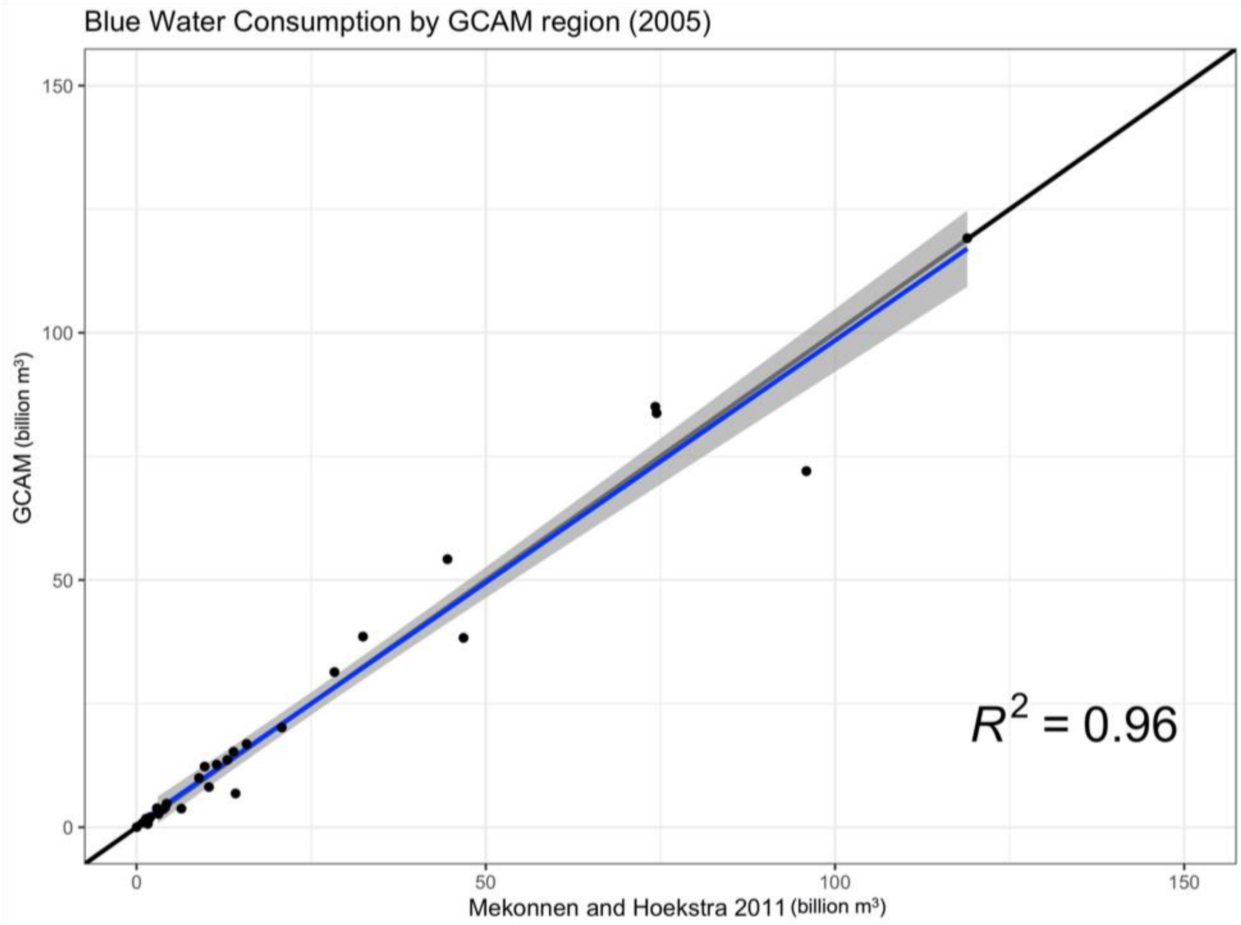

Supplementary Figure 11| Blue water consumption comparison for 2005 values by aggregate GCAM region (points, *n* = 1). Comparison between this study (y-axis), and the Mekonnen and Hoekstra 2011 (x-axis) water footprint of production. Blue line represents a linear fit with 95% confidence interval shown in the grey shading.

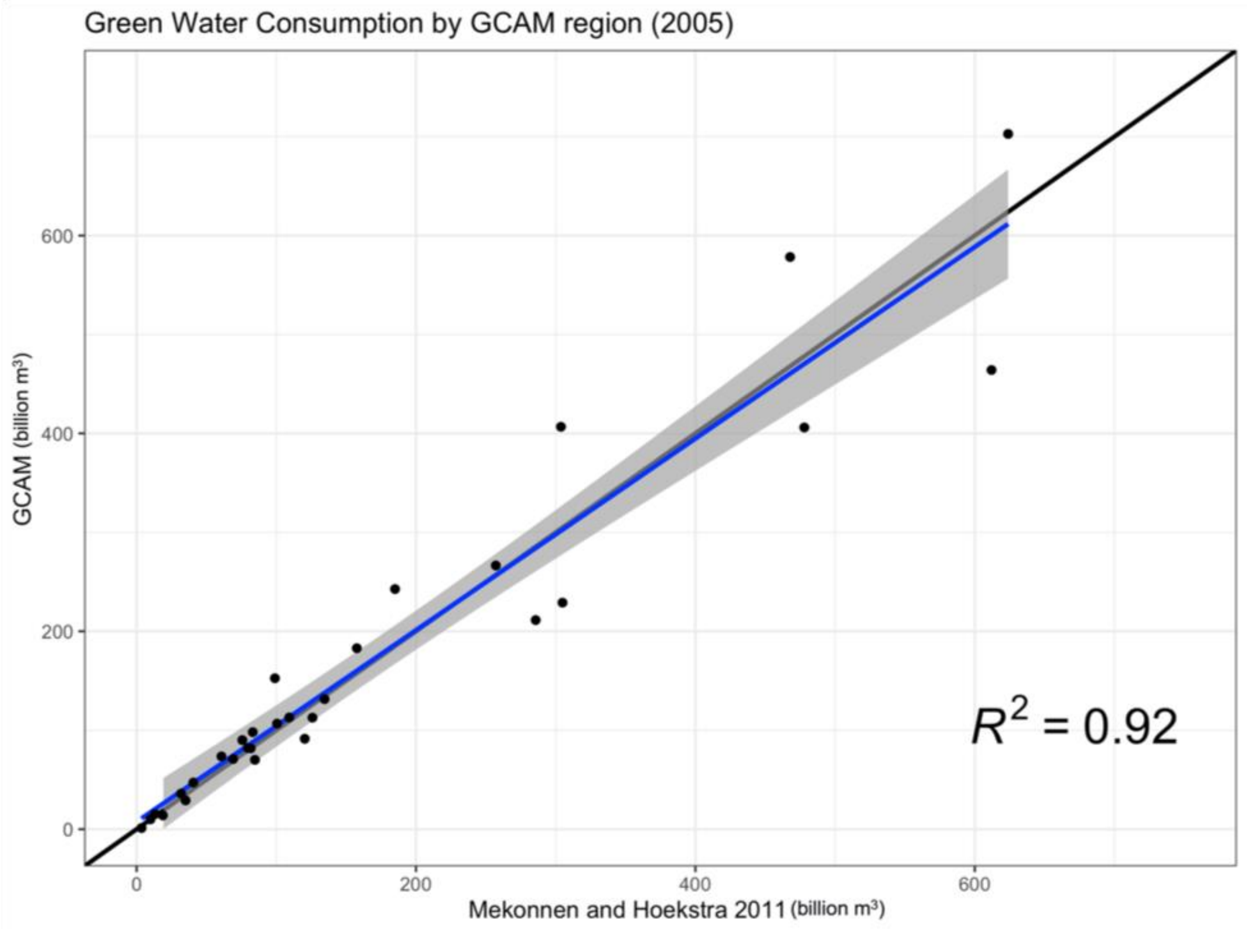

Supplementary Figure 12| Green water consumption comparison for 2005 values by aggregate GCAM region (points, *n* = 1). Comparison between this study (y-axis), and the Mekonnen and Hoekstra 2011 (x-axis) water footprint of production. Blue line represents a linear fit with 95% confidence interval shown in the grey shading.

Supplementary Table 1| Comparison of annual water flows. Comparison to historical and future projected water withdrawals, consumptions, and nonrenewable groundwater depletion through the end of the century.

Water Flows	Annual flows (billion m^3/year)			Source
	2000-2010	2050	2100	
Nonrenewable Groundwater Depletion	280			Wada et al. (2010)
	140			Konnikow (2011)
	292			Dalin et al. (2017)
	332	775		Yoshikawa et al. (2014)
	550	150-1750	60-1500	Kim et al. (2016)
	300		510-680	Wada and Bierkens (2014)
		320-910	110-480	Turner et al. (2019)
	218	520-873	314-401	This Study – SSP2-RCP6.0
Blue Water Withdrawals	3853			FAO (2016)
	4000	5750	6000	Wada and Bierkens (2014)
	3710	6195-8690	4869-12693	Hejazi et al. (2014)
	3250	3700-4200		Bijl et al. (2018)
	3594	4931-5125		Alcamo et al. (2007)
	3860	4875-5120	4490-4820	This Study – SSP2-RCP6.0
Blue Water Consumption	2100			Shiklomanov 1999
	1025			Hoekstra and Mekonnen (2012)
	1300			Döll (2009)
	1690			Hanasaki et al. (2010)
	1772[a]	1656[a]		Pfister et al. (2011)
	1358[a]	2355[a]		Yoshikawa et al. (2013)
	1970		2560-3120	Wada and Bierkens (2014)
	1007[a]	1280-1440[a]	1260-1390[a]	This Study – SSP2-RCP6.0

[a] Consumption associated with irrigation practices alone

Supplementary References

1. Wada, Y., Van Beek, L. P., Van Kempen, C. M., Reckman, J. W., Vasak, S., & Bierkens, M. F. (2010). Global depletion of groundwater resources. Geophysical research letters, 37(20).
2. Konikow, L. F. (2011). Contribution of global groundwater depletion since 1900 to sea-level rise. Geophysical Research Letters, 38(17).
3. Dalin, C., Wada, Y., Kastner, T., & Puma, M. J. (2017). Groundwater depletion embedded in international food trade. Nature, 543(7647), 700-704.
4. Yoshikawa, S., J. Cho, H. G. Yamada, N. Hanasaki, and S. Kanae. "An assessment of global net irrigation water requirements from various water supply sources to sustain irrigation: rivers and reservoirs (1960–2050)." Hydrology and Earth System Sciences 18, no. 10 (2014): 4289-4310.
5. Kim, S. H., Hejazi, M., Liu, L., Calvin, K., Clarke, L., Edmonds, J., ... & Davies, E. (2016). Balancing global water availability and use at basin scale in an integrated assessment model. Climatic Change, 136(2), 217-231.
6. Wada, Y., & Bierkens, M. F. (2014). Sustainability of global water use: past reconstruction and future projections. Environmental Research Letters, 9(10), 104003.
7. FAO (2016). FAO's information system on water and agriculture.
8. Turner, S. W., Hejazi, M., Yonkofski, C., Kim, S. H., & Kyle, P. (2019). Influence of Groundwater Extraction Costs and Resource Depletion Limits on Simulated Global Nonrenewable Water Withdrawals Over the Twenty-First Century. Earth's Future, 7(2), 123-135.
9. Hejazi, M., Edmonds, J., Clarke, L., Kyle, P., Davies, E., Chaturvedi, V., ... & Moss, R. (2014). Long-term global water projections using six socioeconomic scenarios in an integrated assessment modeling framework. Technological Forecasting and Social Change, 81, 205-226.
10. Bijl, D. L., Biemans, H., Bogaart, P. W., Dekker, S. C., Doelman, J. C., Stehfest, E., & van Vuuren, D. P. (2018). A global analysis of future water deficit based on different allocation mechanisms. Water Resources Research, 54(8), 5803-5824.
11. Alcamo, J., Flörke, M., & Märker, M. (2007). Future long-term changes in global water resources driven by socio-economic and climatic changes. Hydrological Sciences Journal, 52(2), 247-275.
12. Shiklomanov, I. A. (1999). World water resources and their use. SHI/UNESCO, St. Petersburg, Russia.
13. Döll, P., Fiedler, K., & Zhang, J. (2009). Global-scale analysis of river flow alterations due to water withdrawals and reservoirs. Hydrology and Earth System Sciences, 13(12), 2413.
14. Hanasaki, N., Inuzuka, T., Kanae, S., & Oki, T. (2010). An estimation of global virtual water flow and sources of water withdrawal for major crops and livestock products using a global hydrological model. Journal of Hydrology, 384(3-4), 232-244.
15. Hoekstra, A. Y., & Mekonnen, M. M. (2012). The water footprint of humanity. Proceedings of the national academy of sciences, 109(9), 3232-3237.
16. Pfister, S., Koehler, A., & Hellweg, S. (2009). Assessing the environmental impacts of freshwater consumption in LCA. Environmental science & technology, 43(11), 4098-4104.

17. Yoshikawa, S., J. Cho, H. G. Yamada, N. Hanasaki, A. Khajuria, and S. Kanae. "An assessment of global net irrigation water requirements from various water supply sources to sustain irrigation: rivers and reservoirs (1960–2000 and 2050)." Hydrol. Earth Syst. Sci. Discuss 10, no. 1 (2013): 1251-1288.
