# Supplementary material for: Future changes in the trading of virtual water
Source: Nat Commun. 2020 Jul 20;11:3632. doi: 10.1038/s41467-020-17400-4 (PMC7371698; doi:10.1038/s41467-020-17400-4)
Supplement: Supplementary file 3 — Reporting Summary [file 41467_2020_17400_MOESM3_ESM.pdf]

# nature research

Corresponding author(s): Neal Graham

Last updated by author(s): Jun 5, 2020

# Reporting Summary

Nature Research wishes to improve the reproducibility of the work that we publish. This form provides structure for consistency and transparency in reporting. For further information on Nature Research policies, see Authors & Referees and the Editorial Policy Checklist.

## Statistics

For all statistical analyses, confirm that the following items are present in the figure legend, table legend, main text, or Methods section.

| n/a | Confirmed | |
|---|---|---|
| ☒ | ☐ | The exact sample size (*n*) for each experimental group/condition, given as a discrete number and unit of measurement |
| ☒ | ☐ | A statement on whether measurements were taken from distinct samples or whether the same sample was measured repeatedly |
| ☒ | ☐ | The statistical test(s) used AND whether they are one- or two-sided<br>*Only common tests should be described solely by name; describe more complex techniques in the Methods section.* |
| ☒ | ☐ | A description of all covariates tested |
| ☒ | ☐ | A description of any assumptions or corrections, such as tests of normality and adjustment for multiple comparisons |
| ☒ | ☐ | A full description of the statistical parameters including central tendency (e.g. means) or other basic estimates (e.g. regression coefficient) AND variation (e.g. standard deviation) or associated estimates of uncertainty (e.g. confidence intervals) |
| ☒ | ☐ | For null hypothesis testing, the test statistic (e.g. *F*, *t*, *r*) with confidence intervals, effect sizes, degrees of freedom and *P* value noted<br>*Give P values as exact values whenever suitable.* |
| ☒ | ☐ | For Bayesian analysis, information on the choice of priors and Markov chain Monte Carlo settings |
| ☒ | ☐ | For hierarchical and complex designs, identification of the appropriate level for tests and full reporting of outcomes |
| ☒ | ☐ | Estimates of effect sizes (e.g. Cohen's *d*, Pearson's *r*), indicating how they were calculated |

*Our web collection on statistics for biologists contains articles on many of the points above.*

## Software and code

Policy information about availability of computer code

| | |
|---|---|
| Data collection | All data was collected study-specific scenarios from computer model runs of the Global Change Analysis Model (GCAM) |
| Data analysis | All analysis was conducted with R version 3.3 or higher |

For manuscripts utilizing custom algorithms or software that are central to the research but not yet described in published literature, software must be made available to editors/reviewers. We strongly encourage code deposition in a community repository (e.g. GitHub). See the Nature Research guidelines for submitting code & software for further information.

## Data

Policy information about availability of data

All manuscripts must include a data availability statement. This statement should provide the following information, where applicable:
- Accession codes, unique identifiers, or web links for publicly available datasets
- A list of figures that have associated raw data
- A description of any restrictions on data availability

The data and code that support the findings of this study are available at https://dx.doi.org/10.25584/1631593 and https://doi.org/10.5281/zenodo.3875735 respectively

# Field-specific reporting

Please select the one below that is the best fit for your research. If you are not sure, read the appropriate sections before making your selection.

☐ Life sciences ☐ Behavioural & social sciences ☒ Ecological, evolutionary & environmental sciences

# Ecological, evolutionary & environmental sciences study design

All studies must disclose on these points even when the disclosure is negative.

| | |
|---|---|
| Study description | This analysis uses GCAM to quantify the amount of water embedded in the global trading of agricultural goods. This water, called virtual water, is calculated based on how much water is consumed by the individual exported crop in the region where it was grown. |
| Research sample | The research sample of this study is direct model output of water consumption, agricultural production, and agricultural demands for each the 32 regions in the Global Change Analysis Model for the year 2015-2100. This data is taken for each of the scenarios described in the main text to test the variability of global circulation model impacts on climate derived agricultural production, water availability, hydropower availability, and building energy requirements. |
| Sampling strategy | The data sampling was taken for each scenario at 5 year intervals from 2015 to 2100. All data was collected from the Global Change Analysis Model dependent upon scenario. |
| Data collection | Data was collected via GCAM model output by the corresponding author |
| Timing and spatial scale | Data for future periods is modeled every 5 years between 2015 and 2100 at either a 235 water basin scale or 32 region scale dependent. No additional data collection was preformed outside of running the GCAM model |
| Data exclusions | No data was excluded from this study |
| Reproducibility | The data of this study, along with analysis code, are available upon request. The Global Change Analysis Model is also a free and open source model available at the following repository: https://github.com/JGCRI/gcam-core |
| Randomization | Randomization was not relevant to this study as each scenario was clearly defined and order of solving would not have changed dependent on scenario. |
| Blinding | Blinding was not relevant to this study as each of the scenarios was run with a computer model with no potential placebo effect. |

Did the study involve field work? ☐ Yes ☒ No

# Reporting for specific materials, systems and methods

We require information from authors about some types of materials, experimental systems and methods used in many studies. Here, indicate whether each material, system or method listed is relevant to your study. If you are not sure if a list item applies to your research, read the appropriate section before selecting a response.

## Materials & experimental systems

| n/a | Involved in the study |
|---|---|
| ☒ | ☐ Antibodies |
| ☒ | ☐ Eukaryotic cell lines |
| ☒ | ☐ Palaeontology |
| ☒ | ☐ Animals and other organisms |
| ☒ | ☐ Human research participants |
| ☒ | ☐ Clinical data |

## Methods

| n/a | Involved in the study |
|---|---|
| ☒ | ☐ ChIP-seq |
| ☒ | ☐ Flow cytometry |
| ☒ | ☐ MRI-based neuroimaging |
